# Haemolysis induced by mechanical circulatory support devices: unsolved problems

**DOI:** 10.1177/0267659120931307

**Published:** 2020-06-23

**Authors:** Inge Köhne

**Affiliations:** Department for Health Services Research, Carl von Ossietzky University of Oldenburg, Oldenburg, Germany

**Keywords:** haemolysis, ventricular assist device, mechanical circulatory support, red blood cell, erythrocyte damage

## Abstract

Since the use of continuous flow blood pumps as ventricular assist devices is standard, the problems with haemolysis have increased. It is mainly induced by shear stress affecting the erythrocyte membrane. There are many investigations about haemolysis in laminar and turbulent blood flow. The results defined as threshold levels for the damage of erythrocytes depend on the exposure time of the shear stress, but they are very different, depending on the used experimental methods or the calculation strategy. Here, the results are resumed and shown in curves. Different models for the calculation of the strengths of erythrocytes are discussed. There are few results reported about tests of haemolysis in blood pumps, but some theoretical approaches for the design of continuous flow blood pumps according to low haemolysis have been investigated within the last years.

## Introduction

The term ‘haemolysis’ refers to the dissolution or degradation of erythrocytes. In the normal life cycle of the red blood cell (RBC), this degradation occurs after about 120 days. The aged and thus inelastic cells are destroyed by mechanical stress and the haemoglobin contained in them is released into the blood plasma and then degraded in the liver, where the iron atoms are recycled. The remains of the cell membrane are absorbed and decomposed by macrophages. In a healthy person, the concentration of free haemoglobin in blood caused by natural haemolysis is about 0.5-2.0 mg/100 L. A level of 10 mg/100 L is currently assumed to be tolerable for humans.^1,2^ If the damage level of RBCs is higher, the production rate of new RBCs cannot substitute the losses. Premature erythrocyte loss can be caused by unnatural haemolysis as a result of increased mechanical stress on the RBC, resulting in increased permeability of the cell membrane for haemoglobin or rupturing of the cell membrane. Haemolysis is also caused by substances that attack the components of the cell membrane, such as soaps, animal toxins and lysing antibodies. Haemolysis can also be caused by excessive osmotic influx of water, which causes the blood cell to burst.^1,3^ The measurement of haemolysis in patients supported by a blood pump (like ventricular assist device, extracorporeal membrane oxygenation (ECMO)) can be impacted by the illness of the patient,^4^ pharmacological management or necessary blood transfusions, so it is not always easy to relate haemolysis to the blood pump alone. The aim of this review is to provide an overview of the current status of research on the strength of erythrocytes and the theoretical assessment of haemolysis in blood pumps. The existing studies about haemolysis in blood pumps are summarized.

## Methods

Electronic searches were performed using PubMed, which includes Medline in April 2019. Search terms were haemolysis and blood pumps. In total, 4,442 articles were found. After title screening and then abstract screening if studies about haemolysis in blood pumps or theoretical approaches concerning haemolysis prediction were described, 93 articles remained for full-text analysis. Forty-eight articles were used for this review and 8 additional ones were found by reviewing the reference lists of these articles.

### Normal Index of Haemolysis

Haemolysis in artificial blood pumps is mainly caused by mechanical stress on the RBCs. The cell walls of the erythrocytes are damaged by shear stresses in the blood, which arise in the blood flow through the movement of blood pumps or through increased wall friction. The damage depends both on the level of the shear stress and on the duration of exposure of the shear stress to the blood cells.^5^ However, the contact of blood cells to foreign materials may induce haemolysis as well.^6^ Another possible reason for haemolysis is low or negative pressure in the flow leading to cavitation, mainly observed during ECMO.^5^ The haemolysis level caused by the individual blood pumps (or other implantations like artificial heart valves, stents etc.) can be described by the Normal Index of Haemolysis (NIH)

NIH[g/100L]=ΔfHbn⋅(1−Ht)⋅ΔN

where ΔfHb is the increase of haemoglobin in blood plasma (mg/dL); Ht is haematocrit (vol.%); blood volume is defined as 1; and ΔN = Qt/V, where Q is the volume flow rate (L/min), t is the duration of the experiment (minutes) and V is the blood volume of experiment (L).^7^

In clinical practice, high haemolysis in patients is detected by increased concentrations of bilirubin and lactate dehydrogenase (LDH) in the blood plasma.^5^

### Haemolysis in blood pumps

There are few published studies on the occurrence of haemolysis in blood pumps. A haemolysis level of 0.19-0.26 g/100 L was measured in vitro in roller pumps.^8^ An in vitro comparison between three centrifugal pumps for extracorporeal short-term support showed that the PediVas pump (Levitronix LLC, Waltham, MA, USA) caused the lowest haemolysis level and the Rotaflow (MAQUET Cardiopulmonary AG, Hirrlingen, Germany) and Medos Deltastream DP3 (MEDOS Medizintechnik GmbH, Stolberg, Germany) about twice as high. It was assumed that the design of the PediVas pump with non-contact magnetic bearings greatly minimizes haemolysis.^9^ The in vitro comparison between Rotaflow and CentriMag (Levitronix LLC) pumps showed a haemolysis level of NIH = 0.021 g/100 L for the Rotaflow and 0.041 g/100 L for the CentriMag.^10^ The in vitro comparison between Biomedicus BP50 (Medtronic BioMedicus Inc., Eden Prairie, MN, USA) and Rotaflow showed advantages for the Rotaflow pump.^11^ Computational fluid dynamics (CFD) analyses in HeartMate II (Thoratec Corp., Pleasanton, CA, USA) and HeartWare HVAD (HeartWare Inc., Framingham, MA, USA) pumps showed no significant difference in the occurring shear stresses. The areas with the highest risk of haemolysis are all gaps between moving and stationary parts of the pumps, as well as the inflow area of the blades of impellers or diffusers. The HeartMate II is an axial pump with a flow straightener, an impeller and a diffusor with three blades each. The impeller is mounted with cup-socket ruby bearings, with a gap of 100 µm each. The diameter of the impeller is 12 mm. The HeartWare ventricular assist device (HVAD) is a centrifugal pump whose impeller with a diameter of 34 mm is magnetically and hydrodynamically mounted. The bearing gaps are 160 µm at the bottom and 40 µm on the top. The maximum shear stress in laminar flow ranges is 200 Pa, the maximum stress in turbulent flow ranges in HVAD Reynolds shear stress is 200 Pa and the maximum shear stress in HeartMate II is 500 Pa.^12^ The flow of the centrifugal pump EVAHEART (EVAHEART Inc. Houston, TX, USA) produces less than half the shear stress of HeartMate II.^13^

The fundamental numerical investigations of haemolysis in axial flow pumps have shown that these pumps have their highest efficiency at an impeller outlet angle of 25°. Impellers with two blades work most effectively at an optimum speed of 6,000 L/min. The critical haemolysis level of this impeller configuration is only exceeded at speeds above 7,000 L/min.^2^ Corresponding investigations in centrifugal pumps show that these have a considerably higher efficiency than axial pumps. The most favourable outlet angle of the impeller blades for these pumps is either 15° or 30°. The highest efficiency is achieved with six blades, and the optimum operating point is four or five blades. The diameter of the outflow tube also has an influence on the efficiency. The optimal pump must be a compromise between effectiveness, pumping capacity and low haemolysis.^14^ The lower the number of blades and the smaller the exit angle, the lower the haemolysis level. In blood pumps with a hydrodynamic radial bearing, haemolysis can be minimized by enlarging the bearing gap. Further influences on the haemolysis level are the bearing diameter, the gap length, the circumferential speed and the pressure difference between inflow and outflow.^15^ When designing a hydrodynamic axial bearing, the geometry of the grooves in the counter-plate of the bearing in the pump casing proved to be decisive for the haemolysis level. By means of an appropriate design, a larger bearing gap is created when the rotor rotates so that the shear load on the blood is reduced and thus also haemolysis. The NIH was reduced by 90% in vitro.^16^

In pulsatile ventricular assist devices (VADs), haemolysis is generally lower because flow velocities and shear rates are lower. Xu et al.^17^ were able to demonstrate that haemolysis in these systems also depends on the operating mode of the pump. In vitro the haemolysis level increases with higher impact rate, higher impact volume and operation with counter-pulsation. However, all these measures have positive effects in reducing the risk of thrombus formation, as the pumps and cannulas are washed out better. The highest stress load on the erythrocytes occurs during the flow through artificial heart valves, where Reynolds shear stresses of up to 1,000 Pa were measured. Measurements in the LionHeart (Arrow International Inc., Reading, PA, USA) measured maximum Reynolds shear stresses of 100 Pa in the blood chamber and 200 Pa in the discharge cannula.^18^

In patients who received a short-term VAD with an IMPELLA^®^ pump (ABIOMED Inc., Danvers, MA, USA), haemolysis was highly dependent on the patient’s disease. There were also many patients without a measurable haemolysis.^4^ This shows that blood pumps, which normally work without serious blood damage, can sometimes cause considerable problems for patients with pre-existing damage. Kusters at al.^19^ have demonstrated by in vitro experiments that an increase in the temperature of the blood has no influence on the haemolysis level. In the in vitro study by Kameneva et al.,^20^ on the contrary, a strong lowering of the temperature (hypothermia) results in a reduction of the deformability of the erythrocytes. Lai et al.^21^ were able to demonstrate in vitro that the use of newly developed superhydrophobic surfaces, also known as the lotus effect, allows the haemolysis level to be reduced for all areas in contact with blood since the shear stresses on these surfaces are greatly reduced. To create these surfaces, a commercially available coating (Rust-Oleum NeverWet) of micro- and nanoparticles with a thickness of 53 µm and a roughness of 2.3 µm was used. In experiments with pulsatile pumps, haemolysis was reduced by 30%. The amount of LDH and bilirubin level in the blood is determined to detect the occurrence of haemolysis in patients with VAD. As an alternative, simple non-invasive procedure, the counting of micro-emboli using transcranial ultrasound Doppler detection (TCD) has proven to be effective. Normally used to detect thromboembolic events, a high number of micro-embolies can indicate haemolysis.^22^

### Non-Newtonian flow behaviour of blood

**Figure 1.**
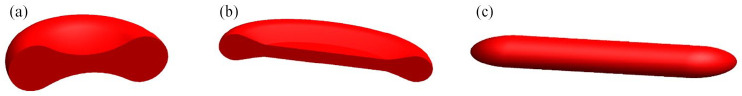
With increasing shear rate, the erythrocytes will be deformed from their (a) natural rotationally symmetrical disc form into an (b) elliptical shape and (c) then into a stretched form before being destroyed. Adapted from Simmonds et al.^5^

**Figure 2.**
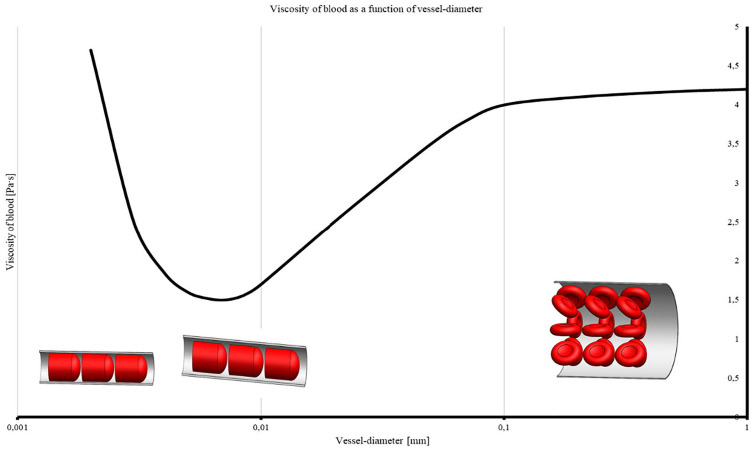
The apparent (measurable) viscosity of blood shows a minimum at a vessel diameter of around 6-8 µm (0.006-0.008 mm). The schematic pictures illustrate the scale between erythrocyte and vessel diameter in different scopes of vessel diameter (logarithmic). Adapted from Pries et al.^3^

For fluids with Newtonian flow behaviour, the viscosity η does not depend on shear stress (τ = η·dv_x_/dy), but only on temperature. Non-Newtonian fluids, on the contrary, do not behave according to Newton’s shear stress approach, but often exhibit structural viscous properties. This means that with increasing shear stress τ, the dynamic viscosity η decreases. This must be taken into account when simulating blood flows in narrow capillaries or with high shear loads.^23^ The viscosity of the blood is mainly determined by the haematocrit and also by the viscosity of the blood plasma, which in turn depends on the concentration of the plasma protein. The deformability and the aggregation tendency of the erythrocytes are a cause for the non-Newtonian flow behaviour of the blood. In contrast to water, blood viscosity therefore depends not only on temperature but also on flow conditions. Because the viscosity of the blood is relatively low when high shear stresses act on the blood in a fast flow but increases considerably in a slow flow and low shear stress, it is referred to as the apparent viscosity of the blood (structure-viscous behaviour). Due to their pronounced deformability, the erythrocytes tend to orient themselves in a blood vessel towards the centre of the flow. With increasing shear rate, the RBCs turn first into elliptical and then stretched shapes before being destroyed (Figure 1). This results in a marginal flow with low cell count which, as a ‘lubricating’ boundary layer (plasma without blood cells), reduces the frictional resistance on the vessel walls (Fåhraeus–Lindqvist effect). The thickness of this boundary layer increases relatively antiproportionally to the reduction of the cross section of the vessels. Therefore, the apparent viscosity of the blood decreases when the vessel diameter drops below 300 µm, reaches a minimum at a diameter of about 6-8 µm and increases again in even narrower capillaries, because the deformability of the erythrocytes now becomes the limiting factor for the friction resistance (Figure 2).^3^ Principally, there is no difference in the flow behaviour of blood in round vessels or in small gaps found in rotary blood pumps. Bearing gaps in these pumps differ between 40 µm (HeartWare HVAD), 250 µm (DuraHeart) and 1 mm (HeartMate III).

### Resistance of erythrocytes

Most of the stress load limits for haemolysis given in the literature refer to shear stress (τ) in the blood and thus to the erythrocytes and are triggered by the flow through mechanical components. It has been shown that the occurrence of haemolysis by shear stress depends not only on the shear stress level but also on the duration of the shear stress.^24,25^ Studies have shown that blood is transported in a laminar flow in all vessels throughout the human body. The highest measured speed is 1,175 mm/s in the femoral artery with a diameter of 5 mm. A turbulent flow only occurs in the aorta during systole with a Reynolds number of Re = 3,000-6,000.^6^ Other potential areas that can cause turbulent blood flow are pathological changes in blood vessels such as plaques, dilations, stenoses and so on.

In experiments, the shear stress varies with different measuring methods so that the test results can only be compared within certain limits, nor can the shear stress be easily determined using Newton’s shear stress approach. Tests have shown that the shear stress only increases linearly at a shear rate of γ >10 s^−1^. The shear rate is the slope of an applied tangent to the flow profile of a laminar flow, calculated with γ = dv_x_/dy. The highest shear rate always occurs directly on the vessel wall, and the highest measured value is 1,885 s^−1^, also in the femoral artery. Goldsmith and Turitto^6^ have determined these deviations in extensive comparative tests. Wu et al.^26^ were able to show that the critical limit of shear stresses determined with a frequently used Couette viscometer is only of limited significance for the occurrence of haemolysis. Beissinger and Laugel^27^ were also able to demonstrate that the haemolysis level increases when the erythrocytes get into contact with foreign materials, regardless of the shear rate present. The presence of free radicals in the blood, which can have an oxidative effect on the erythrocytes, has also reduced their deformability.^28^

The erythrocytes of bovine blood or even porcine blood often used in experiments are smaller than those of human blood. This influences not only the viscosity but also the haemolysis level, because the strength of bovine erythrocytes is higher than that of human erythrocytes. This must be taken into account when estimating haemolysis in such experiments. On the other hand, the erythrocytes of females are more sensitive than those of males.^29–32^

**Figure 3.**
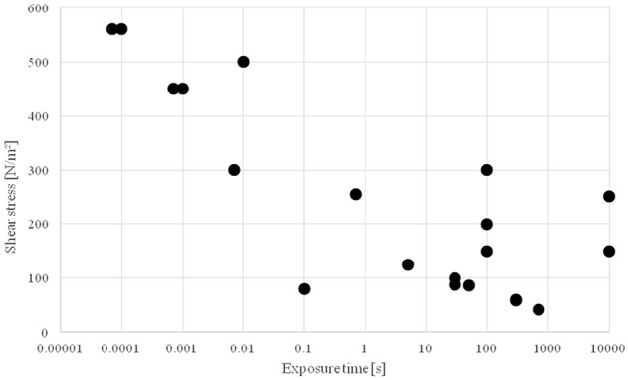
Critical shear stress in laminar flow for one-time exposure. If the shear stress is exceeded, then the erythrocytes will be destroyed. Data from Table 1.

Table 1 summarizes some test results of various research groups for the critical shear stress as a function of the exposure time for laminar flow. The critical shear stress ranges from 42 to 250 Pa for a very long stress exposure time (>10 minutes) up to 560 Pa for a stress exposure time of 0.00007 seconds. Below these shear stress loads, no measurable haemolysis should occur. Figure 3 shows the results graphically. It should be noted that the test results for the critical shear stress were obtained with different test setups for generating the shear stress and with different erythrocyte preparations. The results of the respective tests are all only valid for a one-time stress load on the blood cells. However, the individual test results show a strong scatter so that no real shear stress limit below which no haemolysis occurs can be determined.

**Table 1.** Critical shear stress in laminar flow, which causes erythrocyte destruction if the exposure time is exceeded.

Critical shear stress (Pa)	Exposure time (s)	Reference
560	0.0001	Williams et al.^33^
560	0.00007	Williams^25^
500	0.01	Williams^25^
450	0.0006	Williams^25^
450	0.001	Rooney^34^
300	0.007	Morsink et al.^24^
300	120	Nevaril et al.^35^
255	0.7	Schima et al.^36^
255	0.7	Morsink et al.^24^
150-250	No time limit	Sutera^37^
100-200	100	Sutera et al.^38^
170	100	Schima et al.^36^
150	120	Williams^25^
150	120	Hellums and Brown^39^
150	No time limit	Morsink et al.^24^
125	5	Williams^25^
100	25	Williams^25^
88	40	Williams^25^
86	60	Williams^25^
80	0.15	Giersipen et al.^40^
60	300	Williams^25^
59	300	Williams^25^
42	600	Williams^25^

Summary of results of different investigators.

A flow is called laminar if the individual particles move in parallel paths. In turbulent flows, additional velocity components in all three main directions are superimposed on the main flow. A turbulent flow can occur when the Reynolds number Re exceeds its critical value

Re=υ⋅d/v

where υ is the kinematic viscosity of fluid, d is the diameter of tube and v is the flow velocity.

In circular tubes, the critical Reynolds number is Re = 2,320.^41^ In the human body, a flow with such a high Reynolds number occurs only in the aorta during systole.^6^ After the first artificial heart valves were inserted into human hearts, a flow analysis showed that local hemodynamic conditions downstream of the valves cause turbulences which lead to an increased haemolysis.^40,42^ The ball valves initially used as heart valves (such as Starr-Edwards caged-ball valve) were therefore very quickly replaced by single disc valves, the best known of which was the Björk-Shiley heart valve. Since these valves had permanent fractures in the metal temples,^43^ they are today replaced by double-wing heart valves, such as the St. Jude Medical Regent bileaflet valve. Nevertheless, there are patients who still live with ball valves without complaints. The record is about 50 years.^44^

The shear stress in turbulent flow is called Reynolds shear stress. It is defined as

$$\tau_{Re}=-\rho \times \bar{uv}$$

where ρ is the density of fluid and $\bar{u}\bar{v}$ are the fluctuation velocities in flow.

Since the rates of fluctuation velocities in flow speed $\bar{u}\bar{v}$ could only be measured with the use of laser Doppler anemometry (LDA), the determination of Reynolds shear stress was previously not possible.^45^ It should be noted that the Reynolds shear stress is not a real physical stress, but a pure mathematical quantity resulting from the Navier–Stokes equation of motion in flows. Experiments show a correlation between Reynolds shear stress and haemolysis, but this correlation is not yet evidence of dependency. This is able to explain the partly strong deviations between the test results.^46^ The stress load times of less than 1 ms stated in the test results (Table 2) are also only pure calculation times, because in reality they cannot be measured. The test results of Sallam and Hwang^45^ alone were corrected several times by other research groups through a modified calculation.^46,49,50,53^ Szwast et al.^54^ propose as an alternative to haemolysis risk assessment the determination of energy dissipation (points where flow energy is lost), which can be determined by large eddy flow simulation (LES), a special form of CFD. The Food and Drug Administration (FDA) has now launched an initiative to standardize the application of CFD to the assessment of haemolysis in blood pumps.^55^

**Table 2.** Critical shear stress in turbulent flow (theoretically calculated Reynolds shear stress), which causes erythrocyte destruction if the exposure time is exceeded.

Critical shear stress (N/m²)	Exposure time (s)	References
4,000	0.000001	Forstrom^47^
3,000	Very short	Schima et al.^36^
3,000	Very short	Jhun et al.^46^
1,800	0.00001	Tamagawa et al.^48^
800	0.00001	Lu et al.^49^
600	0.00001	Grigioni et al.^50^
517	0.00001	Yen et al.^51^
400-500	Very short	Schima et al.^36^
400	0.0001	Sallam and Hwang^45^
400	100	Schima et al.^36^
250	240	Sutera and Mehrjardi^52^
60	0.000012	Yen et al.^51^

Summary of results of different investigators.

**Figure 4.**
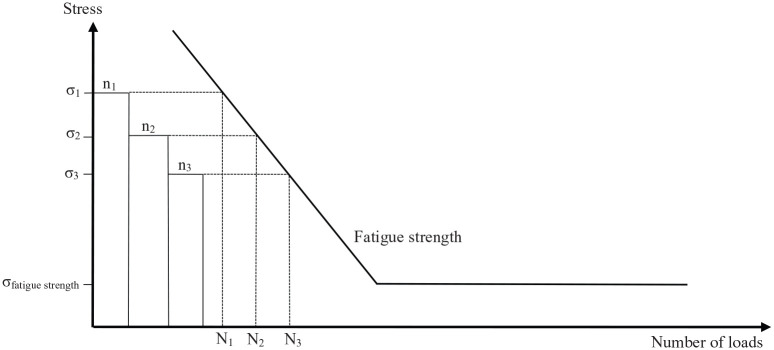
Fatigue strength diagram (stress against frequency of strain = number of loads) as base for the calculation of fatigue strength according to the Miner hypothesis. The sum of loads (represented by rectangles n_1_, n_2_, n_3_) has to be beneath the limit of fatigue strength, that is, a mechanical part (erythrocyte) is in a safe condition.

The investigations considered so far all referred to a single exposure of the erythrocytes. However, this single exposure is not meaningful enough for a prediction of haemolysis in blood pumps since all blood cells have to pass through this blood pump many times a day. As a possible solution, Bludszuweit^56^ has therefore transferred the proven concept of fatigue strength according to the Miner hypothesis, known from technical construction mechanics, to the stressing of blood cells. The idea of calculating the fatigue strength according to the Miner hypothesis is to calculate with a finite number of load cycles. Stresses above the fatigue strength are deliberately permitted because it is no longer assumed that a technical component is safe, but rather that it is likely to fail. The individual stress loads are subdivided into so-called load collectives depending on the load level, which are summed up (Figure 4). From the curve of the fatigue strength of the material from which the component is made, the permissible number of cycles (number of loads with the stress of this load cycle collective) can be read, which are still permissible. According to the Miner hypothesis, a component is considered safe to operate if the sum of all partial damages is D <1

$$D=\sum_{i}\frac{n_{i}}{N_{i}}\leq 1$$

where n_i_ is number of exposures with stress σ_i_ and N_i_ is the maximum admissible number of exposures with stress σ_i_.^57^

For the application of the Miner hypothesis, the triaxial stress state acting on the individual erythrocytes must be converted into a scalar stress value. The conversion to Mises generally employed in construction technology was used^58^

$$\sigma_{scalar}={\left[\frac{1}{6}\cdot \sum^{\;}{\left(\sigma_{ii}-\sigma_{jj}\right)}^{2}+\sum^{\;}\tau_{ij}^{2}\right]}^{\frac{1}{2}}$$

**Figure 5.**
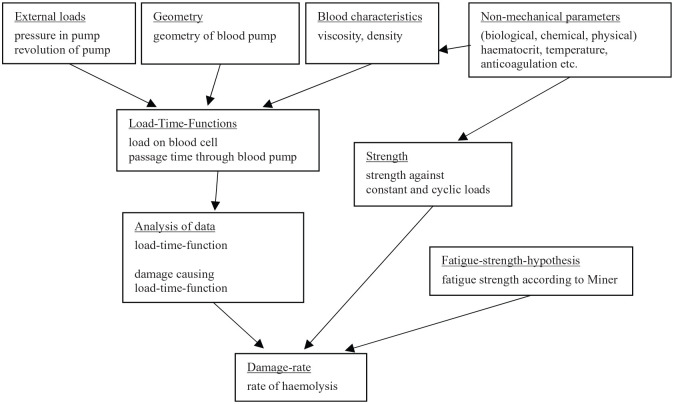
Diagram of process to determine haemolysis in a blood pump. Adapted from Bludszuweit.^56^

Using a radial pump as an example, the complexity of the influences that have to be taken into account for predicting damage to erythrocytes was demonstrated. In addition to the geometry of the pump, the pressure generated by the pump, the peripheral speed generated by the rotational speed, as well as a variety of non-mechanical parameters have an effect. These are blood properties such as density, viscosity, haematocrit and temperature, as well as additional chemical influences through medication or diet. The interaction of all these factors leads to blood damage (haemolysis), which should be assessed. Figure 5 illustrates the relationship between the individual influences for the estimation process.^56^

The Cell Damage Index introduced by Gusenbauer et al.^59^ is intended to sum up the partial damage of the erythrocytes and thus predict haemolysis under multiple stress in CFD models of blood pumps. Chan et al.^32^ have determined the following haemolysis rates (NIH) in experiments of human blood at a stress load of more than 15 minutes (Table 3). These data can be used to compare results from calculations with CFD models.

**Table 3.** Haemolysis level (NIH) depending on shear rate after Chan et al.^32^

Shear rate (s^−1^)	NIH (mg/dL)
2,000	40
4,000	70
6,000	90
8,000	170

NIH: Normal Index of Haemolysis.

Another limit for haemolysis is the maximum permissible surface tension in the cell membrane of the erythrocytes. This is indicated between σ = 0.008 N/m^60^ and σ = 0.01-0.03 N/m.^61^ The cell membrane of the erythrocytes represents an interface, that is, a separating surface between two different phases (fluid regions). The energy content of the molecules inside the cell and on the surface of the cell is different. Curved membranes (interfaces) lead to pressure differences at which the higher pressure prevails inside the cell. This pressure difference causes an interfacial tension (surface tension) in the membrane. There are various theories for calculating the membrane stress. Erythrocytes in a viscous fluid deform when subjected to hydrodynamic shear stress. The cells assume an ellipsoidal shape and align themselves parallel to the flow. The cell membrane of the erythrocytes rotates around the interior of the cell like the caterpillar track on a tracked vehicle. This exposes the entire cell membrane to the maximum velocity gradient dv_x_/dy. The deformation of the cell increases continuously with increasing shear stress until a limit is reached where the surface of the ellipsoid is just large enough to enclose the incompressible cell interior with haemoglobin. If the shear stress increases further, the cell cannot stretch further, so the membrane is tangentially stretched. If the critical limit of the membrane tension is exceeded, the cell membrane tears and the erythrocyte is destroyed. In the calculation model from Williams,^25^ the membrane stress results in

$$\sigma =\frac{\tau \cdot r\cdot 1.017}{\left(L-B)/(L+B\right)}$$

where r is the radius of non-deformed erythrocyte, L is the length of deformed erythrocyte and B is the breadth of deformed erythrocyte.

If a viscoelastic behaviour is assumed for the membrane of the erythrocytes, which can be illustrated by a mechanical model consisting of two spring and two viscous damper systems, an equation for the critical membrane stress can be assumed to be

$$\frac{1}{\sigma_{critical}}=\frac{1}{\varepsilon_{critical}\cdot d}\cdot \left[\frac{1}{Y_{2}}+\frac{1}{Y_{1}}\cdot \left(1-e^{-\frac{Y_{1}}{\eta_{1}}\cdot t}\right)+\frac{t}{\eta_{2}}\right]$$

where e_critical_ is the critical extension of erythrocyte, d is the thickness of membrane, t is the exposure time of shear stress, Y_1_ = 27-210 N/cm², Y_2_ = 11-93 N/cm², η_1_ = 5.0 × 10^7^-5.2 × 10^8^ Pa s and η_2_ = 3.7 × 10^8^-3.1 × 10^9^ Pa s.

In the calculation model of Richardson,^62^ the membrane stress results from

$$\sigma =1.7\cdot \gamma \cdot \eta \cdot \frac{a}{d}\cdot \sin(2\cdot \chi )$$

where γ is the shear rate, η is the dynamic viscosity of blood plasma, a is the large ellipsoid radius of erythrocyte, d is the thickness of membrane and χ is the angle of rotation of erythrocyte.

Experiments with human erythrocytes, which were subjected to individual mechanical loads, have shown that the deformation behaviour is predominantly elastic. A viscoelastic deformation could only be observed at very high stress load frequencies. However, such high frequencies do not occur in real blood flows.^63^ Thus, the two previous models are not suitable for an estimation of the membrane stress. Sohrabi and Liu^64^ have developed a computer model of an erythrocyte whose behaviour under simulated stress should also reflect permanent strength. A similar virtual model is also available from Toninato et al.^65^ All models for calculation of haemolysis in CFD are either stress-based or strain-based. They rely on either a Lagrangian model or the Eulerian transport equations. In Lagrangian models, haemolysis is calculated along pathlines of the flow. In the more complex Eulerian approaches, the transport equations have to be solved within the CFD, using Navier–Stokes and turbulence equations. The power-law model first invented by Giersiepen et al.^66^ also calculates haemolysis as the increase in free haemoglobin in the blood to the total haemoglobin in the erythrocytes depending on the shear stress and exposure time

$$\frac{\Delta \mathrm{Hb}}{\mathrm{Hb}}=C\cdot \tau^{-\alpha }\cdot t^{\beta }$$

where ΔHb is the released haemoglobin; Hb is the total haemoglobin in erythrocytes; τ is the shear stress (scalar); t is the exposure time; and C, α and β are constants

A comparison of the current CFD models for haemolysis can be found in Yu et al.^67^ Since human erythrocytes are rarely used for haemolysis experiments due to their costly and time-consuming procurement, Pohl et al.^60^ have tried to develop artificial erythrocyte models. They consist of long-chain polymer molecules made of polyacrylamide. Comparative tests with human and animal blood have proven the suitability of these artificial erythrocytes for the assessment of haemolysis in fluid flow.

## Discussion

Theoretical investigations and practical experiments on haemolysis and on the strength of erythrocytes have been carried out since 1968. The results were different and do not give a uniform picture. While at the beginning only laminar flows were considered, since the work of Sallam and Hwang^45^ theoretical Reynold shear stresses have also been the focus of the investigations. However, the results are evaluated and interpreted quite differently. The generally cited critical threshold for Reynold shear stress of 400 N/m² has recently been questioned.^46,49,50,51^ Since all experiments referred only to a one-time stress load of the erythrocytes, Bludszuweit^56,58^ in particular dealt with the fatigue strength of the erythrocytes. Various models for the strength of erythrocytes^25,60^ can be considered unrealistic by Puig-de-Morales-Marinkovic et al.’s^63^ tests on individual erythrocytes. In the meantime, there are approaches to estimate the strain on RBCs in CFD simulations using corresponding erythrocyte models. The NIH has established itself as a tool widely used for assessing haemolysis. There are relatively few attempts to determine haemolysis levels in currently used blood pumps. All the short-term VADs examined have a haemolysis level of >0.01 g/100 L, which is currently considered a tolerable limit.^9,10^ However, roller pumps have a haemolysis level that is 10 times higher.^8^ However, these pumps are mainly used in clinical practice for short-term applications with low flow rates, such as dialysis. For ECMO, they are often been replaced by centrifugal pumps that are less critical due to haemolysis. The sixth INTERMACS report with data of 2006-2013 gave an occurrence of haemolysis in those with long-term VAD in 5% of all patients between 2006 and 2010 and in 9% of all patients between 2011 and 2013.^68^ For long-term VAD, only CFD calculations for the occurring shear stresses have been published so far. The results were more favourable for the compared centrifugal pumps than for the axial pump investigated.^12,13^ The latest theoretical work shows how the haemolysis level in axial and centrifugal pumps can be reduced by optimizing design parameters. A reduction of shear stresses in blood pumps will not only reduce haemolysis but also damage other blood components such as platelets, white blood cells or von Willebrand factor. This can help reduce the von Willebrand syndrome and the occurrence of thrombosis and thus increase the survival rate of the patients.

## Conclusion

Since VADs are essentially rotary pumps, haemolysis has once again become the focus of research, as the stress on the erythrocytes in these systems, with their partly turbulent flow, is much higher than in the pulsatile volume displacement pumps. The problem of haemolysis can be greatly minimized by careful consideration of the pump design with regard to blood-sparing flow dynamics. The main areas in these pumps which have to be designed carefully are all small gaps. The use of contactless bearings like magnetic or hydrodynamic bearings will reduce the risk of haemolysis. There are patients today who have lived with these VADs for more than 10 years with almost no problems. Haemolysis generated by VAD should be further reduced, especially for use as destination therapy over long periods of time.
